# Costs of Transmission Assessment Surveys to Provide Evidence for the Elimination of Lymphatic Filariasis

**DOI:** 10.1371/journal.pntd.0005097

**Published:** 2017-02-01

**Authors:** Molly A. Brady, Rachel Stelmach, Margaret Davide-Smith, Jim Johnson, Bolivar Pou, Joseph Koroma, Kingsley Frimpong, Angela Weaver

**Affiliations:** 1 Global Health Department, RTI International, Washington, DC, United States of America; 2 Global Health, Population, and Nutrition (GHPN) Department, FHI360, Washington DC, United States of America; 3 Emerging Markets, Deloitte Consulting, Accra, Ghana; 4 Neglected Tropical Disease Program, United States Agency for International Development, Washington DC, United States of America; Task Force for Child Survival and Development for Global Health, UNITED STATES

## Abstract

**Background:**

To reach the global goal of elimination of lymphatic filariasis as a public health problem by 2020, national programs will have to implement a series of transmission assessment surveys (TAS) to determine prevalence of the disease by evaluation unit. It is expected that 4,671 surveys will be required by 2020. Planning in advance for the costs associated with these surveys is essential to ensure that the required resources are available for this essential program activity.

**Methodology and Findings:**

Retrospective cost data was collected from reports from 13 countries which implemented a total of 105 TAS surveys following a standardized World Health Organization (WHO) protocol between 2012 and 2014. The median cost per survey was $21,170 (including the costs for rapid diagnostic tests [RDTs]) and $9,540 excluding those costs. Median cost per cluster sampled (without RDT costs) was $101. Analysis of costs (excluding RDTs) by category showed that the main cost drivers were personnel and travel.

**Conclusion:**

Transmission assessment surveys are critical to collect evidence to validate elimination of LF as a public health problem. National programs and donors can use the costing results to adequately plan and forecast the resources required to undertake the necessary activities to conduct high-quality transmission assessment surveys.

## Author Summary

As national programs are nearing elimination of lymphatic filariasis as a public health problem, questions have been raised about the cost of collecting the data necessary for documenting validation of elimination. A series of standardized population-based surveys is necessary to determine prevalence of infection in endemic areas. The authors retrospectively collected data on the costs of these surveys from 13 countries to determine median cost per survey and per cluster sampled. Costs were found to be comparable with other neglected tropical disease surveys. The major cost drivers were personnel and travel for supporting collection of data in the field. National programs and donors can use these results to plan and advocate more effectively for sufficient resources to support validation of elimination.

## Introduction

Lymphatic filariasis (LF) is a parasitic disease, transmitted by mosquitoes, found amongst the most poor and vulnerable populations in 73 countries. One of a group of neglected tropical diseases (NTDs), it can cause chronic medical conditions such as the swelling of legs and arms (lymphedema and elephantiasis) and of the scrotum (hydrocele). It is estimated that over 1.1 billion people are at-risk from LF, with 36 million people having chronic complications [1]. The World Health Organization (WHO) aims to eliminate the disease as a public health problem globally by 2020 [2].

National LF programs focus on a series of critical steps following standardized WHO protocols: i) mapping to determine endemicity, ii) implementation of mass drug administration (MDA) once-yearly for at least five years in endemic areas, iii) a survey to determine whether prevalence is low enough for MDA to stop, iv) post-MDA surveillance, and v) validation of elimination of LF as a public health problem. MDA is implemented with albendazole and ivermectin in areas co-endemic for onchocerciasis and with albendazole and diethylcarbamazine (DEC) in areas without onchocerciasis in program-defined implementation units (IUs), often administrative districts.

After at least five years of MDA with effective coverage, defined as coverage of at least 65% of the total population in each IU, and very low or zero levels of microfilaremia (<1%) or antigenemia (<2%) prevalence in sentinel and spot-check sites, WHO recommends implementing a transmission assessment survey (TAS) to determine whether or not to stop MDA [3]. The TAS is a population-based survey which is designed to determine whether the prevalence of the parasite has been reduced to a level below which continued transmission is no longer expected, even in the absence of further interventions. The TAS takes place in an evaluation unit (EU), which can be equivalent to an IU, a combination of IUs with similar epidemiologic characteristics, or part of an IU, depending on population size. In all countries included in this study, besides Vietnam, a cluster sampling methodology was used. For Vietnam, three of the four surveys used a systematic sampling methodology instead, in which they visited every school in the EU. The survey measures the prevalence of antigen (in *W*. *bancrofti* areas) or antibody (in *Brugia* spp areas) in 6 and 7 year olds. This age group is selected as they should be protected from infection if MDA has been successful in interrupting transmission. Circulating filarial antigenemia is detected by the immunochromatographic test (ICT) [Alere Inc., Scarborough, ME] and antibody by the Brugia Rapid test [Reszon Diagnostics International, Subang Java, Selangor, Malaysia], both of which are point-of-care, rapid diagnostic tests (RDTs) that use fingerprick blood to give results in 10–25 minutes.

If the survey finds less than or equal to a critical cut off number, the EU is eligible to stop MDA. The critical cut offs are based on levels of 2% antigenemia/antibody in areas with *Anopheles* or *Culex* vectors and 1% antigenemia in *Aedes* areas. Once MDA is stopped, WHO guidance is to repeat the TAS at 2–3 and 4–6 years after stopping MDA as part of post-MDA surveillance in order to ensure recrudescence is not occurring.

In areas with greater than or equal to 75% net primary school enrolment rate, clusters in TAS are equivalent to schools, usually in 1^st^ and 2^nd^ grades where the majority of students are 6 or 7 years old. In areas with less than 75% enrolment rate, clusters in TAS are census enumeration areas or villages and individual households are sampled.

Given the 4,671 TAS predicted to be necessary to implement by 2020, it is critical to understand the costs of a TAS. National programs need this information in order to develop realistic budgets and understand the financial underpinning of the transition from MDA activities to post-MDA surveillance. At global levels, WHO, implementing partners and donors also need reliable cost data to advocate for additional resources for national programs and to plan their own budgets to support national programs. While the number of TAS needed by year globally can be forecast by WHO, there is little information on the actual costs of TAS surveys. This article provides estimates of the median costs per survey in an EU and per sampled cluster, including an analysis of cost drivers.

## Methods

### Data collection

Data were collected retrospectively from countries which received funding from the U.S. Agency for International Development’s (USAID) NTD Program to support TAS between 2012–2014 under three USAID-supported projects: ENVISION, END in Africa and END in Asia. Support to Ministries of Health was provided through implementing partners, i.e. international non-governmental organizations (NGOs) including FHI360, Health & Development International (HDI), Helen Keller International (HKI), IMA World Health, and RTI International.

USAID’s NTD Program and its projects utilize different financial mechanisms to support country-level implementation of activities. In the six countries where per diem, lodging, travel, and supplies were directly paid by implementing partners, actual expenditure data were collected. In seven countries, grants with local NGOs or the Ministries of Health were used to support survey implementation. These grants are a mechanism by which funding is given to the Ministry of Health or a local NGO based on both a detailed budget review and the meeting of specific grant milestones, but without submission of receipts or other financial records. In countries using the grant mechanisms, the grant budgets were used as a proxy for actual costs.

An Excel-based data collection tool was used to collect the survey costs. Survey costs were divided into categories (supplies, personnel [per diem/lodging], travel, and other) and activities (pre-meetings, training, survey itself, post-meetings, and other) (Table 1).

**Table 1:** Activities used and ingredients included.

Activities	Ingredients
Pre-meetings - To sensitize communities or schools - To plan for surveys with local teams	- Meeting facilities - Supplies - Honoraria for resource persons - Per diem/lodging - Travel
Training - To implement on-the-job training within EUs to train local staff	- Meeting facilities - Supplies - Food - Honoraria for resource persons - Per diem/lodging - Travel
Survey itself - To actually implement survey data collection	- RDTs - Supplies for using RDTs (gloves, lancets, cotton balls, biohazard bags, etc.) - In-country transport of RDTs - Drugs to treat positive survey participants - Social mobilization of communities - Stationery - Per diem/lodging - Travel - Vehicle rental and/or fuel - Prepaid phone cards - Snacks for survey participants
Post-meetings - To inform district health offices of results	- Meeting facilities - Supplies - Honoraria for resource persons - Per diem/lodging - Travel
Confirmatory testing - To test positive children for microfilaremia	- Slides - Lab consumables
Survey design, data analysis, and report writing	- Support to local NGOs for survey design, data collection and report writing
Other	- Ethics review committee fees - Overhead costs for local NGO support - Translation costs - Contingency costs

Data were also collected per survey on population, population density, number of IUs, number of clusters, number of days, and number of RDTs used. Where both household-based and school-based TAS were conducted in the same country and year, they were treated as separate TAS.

The data reflect the incremental costs of TAS and do not include Ministry of Health or international NGO salaries and associated costs or vehicles provided by Ministries of Health. Costs did include support to local NGOs to provide technical assistance or help with transferring and accounting for funds and reporting. Costs did not include support from international NGOs for technical assistance, supervision or transferring funds (e.g. staff time on developing grants or disbursing per diems) as external technical assistance is not required by WHO for a national program to implement a TAS and these costs vary depending on international organization’s policies.

Many countries did host TAS training for central teams or for regions and districts with upcoming TAS. Given the variation in training objectives, participants, funders and involvement of international facilitators among these trainings, these costs were excluded from this analysis.

### Data analysis

Data were reported in USD or in local currency, depending on the accounting system of each implementing partner. When reported in local currency, the average exchange rate for the year in which the survey took place was used to convert the cost to US dollars (USD) (oanda.com). Costs were then converted to 2012 USD using the GDP implicit price deflator (bea.gov).

All surveys financed in the same USAID fiscal year in the same setting (e.g., school or community) were reported together and a mean cost per survey calculated. Data were analyzed in RStudio (Version 3.2.1; RStudio Inc., Boston, MA) using the Wilcoxon rank sum test. Results are presented with the 95% confidence interval (CI) for the difference in location of medians of the two compared groups.

## Results

Twenty cost data collection reports were submitted from 13 countries which conducted TAS with USAID support between 2012 and 2014. Countries and costing sources are outlined in Table 2. Four (20%) reports were from TAS implemented in 2012, seven (35%) in 2013, and nine (45%) in 2014. Eleven came from countries in the WHO Africa Region, seven from countries in the South East Asia Region, and two from countries in the Western Pacific Region. Twelve reports were based on grant budgets, while the other eight were based on actual expenditures incurred by implementing partners. The median number of TAS included in each report was 4 (interquartile range (IQR): 3–6).

**Table 2:** TAS reports included in cost analysis, by country, year and cost source.

Country	Year	Cost Source	Project	Implementing Partner	Number of TAS
Bangladesh	2012	Grant budget	END in Asia	FHI360	7
Bangladesh	2013	Grant budget	END in Asia	FHI360	6
Benin	2014	Actual expenditures	ENVISION	RTI	6
Burkina Faso	2012	Grant budget	END in Africa	HKI	4
Burkina Faso	2013	Grant budget	END in Africa	HKI	2
Burkina Faso	2014	Grant budget	END in Africa	HKI	4
Cambodia	2013	Actual expenditures	END in Asia	FHI360	4
Cameroon	2014	Actual expenditures	ENVISION	HKI/local NGO	3
Ghana	2014	Grant budget	END in Africa	FHI360	24
Indonesia	2012	Actual expenditures	ENVISION	RTI	5
Indonesia	2013	Actual expenditures	ENVISION	RTI	2
Indonesia	2014	Actual expenditures	ENVISION	RTI	5
Nepal	2013	Grant budget	ENVISION	RTI/local NGO	7
Nepal	2014	Grant budget	ENVISION	RTI/local NGO	2
Niger	2014	Grant budget	END in Africa	HKI	5
Tanzania	2014	Actual expenditures	ENVISION	IMA	2
Togo	2012	Grant budget	END in Africa	HDI	3
Uganda	2013	Actual expenditures	ENVISION	RTI	4
Uganda	2014	Actual expenditures	ENVISION	RTI	6
Vietnam	2013	Grant budget	END in Asia	FHI360	4
TOTAL					105

As shown in Table 3, a median of 1.43 IU’s were included in each survey (IQR: 1.00–2.25). The median population per EU was 379,704 people (IQR: 275,555–845,485), with a median population density of 106 people/kilometer squared (IQR: 66–352). A median of 1,653 RDTs were administered per EU (IQR: 1,558–1,718). The median number of days per TAS was 13.4 (IQR: 9.28–20.8).

**Table 3:** Costs of TAS, including or excluding the costs of RDTs (2012 USD).

Country	Year	Setting	No. of TAS	Mean no. of clusters per EU	Mean no. of IU's per EU	Mean population per EU	Mean population density per EU (people/ km^2^)	Mean no. of days per TAS	Mean no. of RDTs per EU	Mean cost per TAS with RDT costs (USD)	Mean cost per TAS without RDT costs (USD)
Bangladesh	2012	School	7	31.1	0.71	1,389,571	1,081	13.7	1692.0	11,878	7,805
Bangladesh	2013	School	6	30.0	0.83	1,147,000	810	8.0	1692.0	15,711	7,690
Benin	2014	School	5	39.2	4.20	456,776	152	10.0	1625.4	13,574	4,671
Benin	2014	Household	1	46.0	4.00	206,656	23	22.0	1612.0	31,159	22,329
Burkina Faso	2012	Household	4	30.5	2.25	307,923	62	13.0	2322.2	37,262	21,587
Burkina Faso	2013	Household	2	30.0	3.00	913,533	65	11.0	4522.0	82,991	28,186
Burkina Faso	2014	Household	4	35.0	2.25	636,190	66	10.5	1500.0	25,682	15,878
Cambodia	2013	School	4	30.0	1.00	273,261	30	16.3	1722.8	13,768	8,891
Cameroon	2014	School	3	32.3	1.67	248,187	54	8.0	1696.3	25,822	12,976
Ghana	2014	School	24	35.8	2.67	414,064	1,057	9.0	1887.0	9,624	5,348
Indonesia	2012	School	5	33.4	1.00	328,942	981	4.4	1490.2	26,122	4,411
Indonesia	2013	School	2	30.5	1.00	282,435	115	5.0	1594.7	26,069	4,676
Indonesia	2014	School	5	31.8	1.00	345,344	97	5.2	4087.0	24,967	6,640
Nepal	2013	School	7	48.4	2.29	1,082,692	357	21.0	1680.6	16,646	7,018
Nepal	2014	School	2	34.0	2.50	1,601,177	338	41.5	1705.0	21,409	12,468
Niger	2014	Household	5	30.0	1.20	641,343	94	22.0	3200.0	33,056	24,795
Tanzania	2014	School	2	36.5	1.00	221,645	96	24.0	1599.5	29,721	20,961
Togo	2012	School	3	30.3	2.00	305,092	97	10.0	1250.0	14,879	9,392
Uganda	2013	School	4	31.0	1.00	243,778	128	16.0	1562.5	8,836	6,820
Uganda	2014	School	3	31.0	1.00	214,165	124	20.3	1546.7	17,382	9,688
Uganda	2014	Household	3	44.3	2.00	424,875	48	23.3	1556.3	20,929	13,187
Vietnam	2013	School	4	20.5	1.00	915,726	642	14.5	900.0	15,408	11,750
**Mean**				**33.7**	**1.71**	**572,744**	**296**	**14.9**	**1929.3**	**23,770**	**12,140**
**Median**				**31.5**	**1.43**	**379,704**	**106**	**13.4**	**1653.0**	**21,170**	**9,540**

### TAS costs

The cost per TAS is presented in Table 3, both with and without the costs of the RDTs. The mean cost per TAS was skewed by outliers (Burkina Faso, Niger and Tanzania), so are described by the median: $21,170 with RDTs and $9,540 without RDTs. Median cost without RDTs per cluster was $101 (IQR $42 - $160).

RDT cost varied depending on which implementing partner procured the tests. For the ICT, the mean cost of one test, including shipping, was $4.54 (range: $2.64, $9.03). For Brugia Rapids, the mean cost was $9.73 when purchased locally. RDT costs accounted for a mean of 46.05% of overall TAS costs (range: 22.82%, 83.11%). The remainder of the costs will be presented as the cost without RDTs.

### Composition of non-RDT TAS costs

Costs are presented by category and by activity as a percentage of overall costs in Table 4. Personnel costs were the highest (50% of the total cost of a survey), followed by travel (34%), supplies (10%), and other (7%). The majority of TAS costs were spent on the survey itself (79%), with small amounts supporting pre- or post-survey meetings (9%), on-the-job training (5%), testing of children with positive results for microfilaremia (1%), and time for design and/or reporting (3%). Not all programs included pre- or post-survey meetings, on-the-job training, or testing of children with positive results as part of TAS implementation. The time of design and/or reporting was only included if a local NGO was engaged to implement these activities.

**Table 4:** Mean cost per TAS, excluding RDTs, as a percentage of total costs.

Activity	Costs per Category				
	Personnel	Travel	Supplies	Other	Total
**Pre-meetings**	4.3%	2.1%	2.5%	0.1%	**9.0%**
**Training**	2.6%	0.9%	1.0%	0.2%	**4.6%**
**Survey itself**	42.4%	30.2%	5.3%	0.8%	**78.8%**
**Post-meetings**	0.1%	0.1%	0.0%	0.2%	**0.3%**
**Confirmatory testing**	0.1%	0.0%	1.0%	0.4%	**1.4%**
**Survey design, data analysis, and report writing**	0.0%	0.0%	0.0%	2.8%	**2.8%**
**Other**	0.2%	0.4%	0.3%	2.1%	**3.0%**
**Total**	**49.8%**	**33.7%**	**10.0%**	**6.6%**	**100.0%**

**Fig 1:**
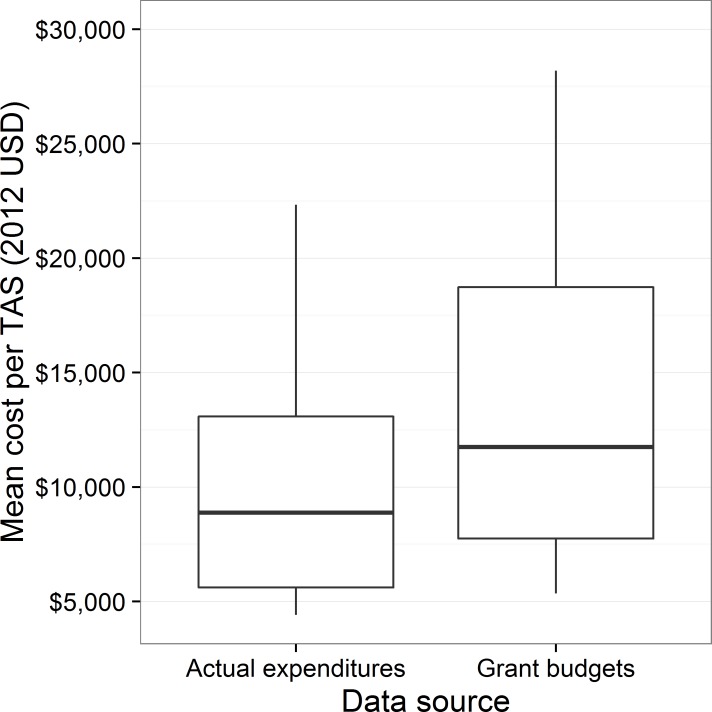
Mean cost per TAS by source of expenditure data.

The mean cost per TAS by the source of the expenditure data is displayed in Fig 1. Whether costs were determined by actual expenditures (based on receipts collected) or by grants (based on negotiated costs) did not appear to be significantly associated with the mean cost per TAS (95% confidence interval (CI): -$1,883, $8,612).

**Fig 2:**
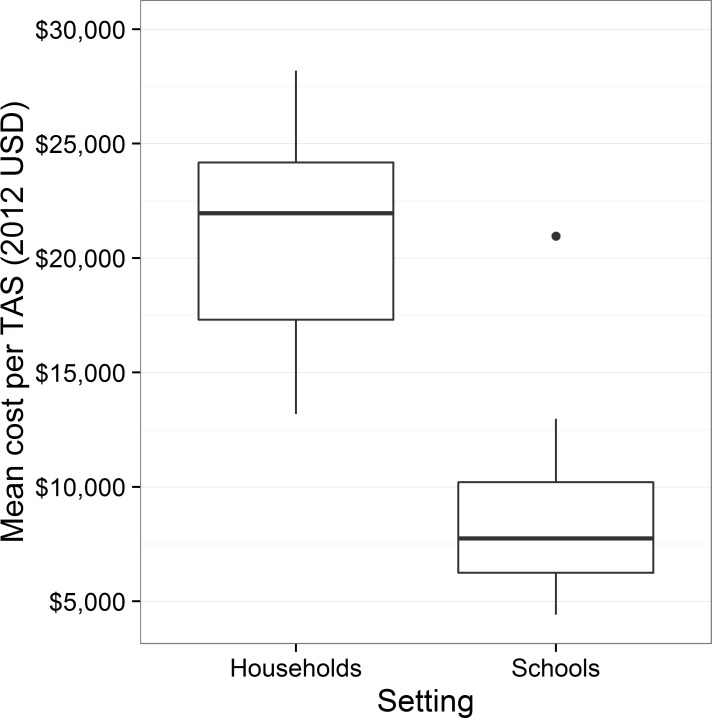
Mean cost per TAS by survey setting.

As shown in Fig 2, school-based surveys had a median cost of $7,748 (IQR: $6,245, $10,203), while household surveys had a median cost of $21,958 (IQR: $17,306, $24,178). Mean costs per TAS of household-based surveys significantly exceeded those of school-based surveys, with a difference in location of $12,817 (95% CI: $6,988, $17,653). School-based surveys were carried out in EUs with a higher population density than household surveys, with a difference in location of 87.88 people per km^2^ (95% CI: 31.63 people per km^2^, 744.94 people per km^2^). They did not differ significantly in the number of days required for overall TAS (95% CI: -12.00 days, 3.50 days),but they did differ significantly in the number of preparation days required before TAS implementation, with household-based surveys requiring more preparation time (difference in location 2.00 days, 95% CI: 1.00 days, 5.00 days).

**Fig 3:**
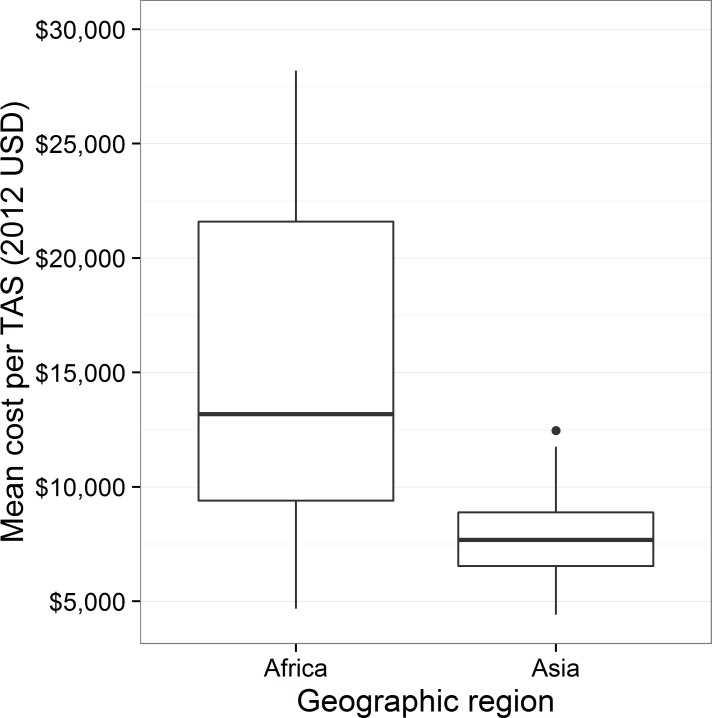
Mean cost per TAS by geographic region.

The median of the mean cost per TAS appeared significantly greater for countries in Africa than in Asia, with a difference in location of $6,169 (95% CI: $508, $14,416) though as shown in Fig 3, the mean cost per TAS varied much more within Africa than within Asia. The median cost of a TAS in Asia was $7,690 (IQR: $6,544, $8,890) versus a median cost in Africa of $7,748 (IQR: $6,245, $10,203). The mean population density for EUs in Asia significantly exceeded those for EUs in Africa, with a difference in location of 272.51 people per km^2^ (95% CI: 7.36 people per km^2^, 744.94 people per km^2^). In addition, all of the household-based surveys occurred in Africa. The regions did not differ significantly in number of days per TAS (95% CI: -5.25 days, 8.26 days).

## Discussion

Since WHO released the new Global Programme to Eliminate Lymphatic Filariasis monitoring and evaluation guidance in 2011, there has been no systematic effort to determine the cost of a TAS to governments and other stakeholders. This analysis of cost data collected from reports from 13 countries shows a median cost per survey in an EU of $21,170 including RDTs and $9,540 excluding RDTs, and a median cost per cluster of $101 excluding RDTs.

Costs reported in this article are similar to those published in a 2011 article by Chen et al on the costs of trachoma prevalence surveys in eight national programs [4]. The authors reported a median cost per district survey of $4,784 and median cost per cluster of $311. Since trachoma surveys are based on clinical diagnosis, don’t include RDTs, and have fewer numbers of clusters per district than TAS, the best comparison with TAS data is the median cost of a TAS cluster excluding RDTs, or $101.

A 2013 article by Chu et al reported on a multicenter evaluation of TAS as part of an operational research project [5]. The authors reported a median cost per survey of $24,900; however, these costs included the costs of international technical assistance, extra specimen collection and shipping, and intensive training related to the research objectives.

Personnel and travel were found to be the major cost drivers in this study. Chen et al and Chu et al likewise found personnel and travel to be the main drivers of survey cost. Many of the surveys were implemented by national teams which traveled to and throughout the survey area to collect data. However, as lower-level (provincial and district) health and laboratory personnel gain experience in implementing TAS, greater efficiencies in travel costs may be found by using more staff from lower levels, with national or regional level supervision. RDTs were not included as a major cost driver, given the variation in costs of RDTs and the fact that a global consortium recently has pledged to support the costs of antigen-based diagnostic tests for TAS.

However, given that costs for on-the-job training and design and reporting were difficult to separate from survey costs, it is likely that some costs captured under the survey itself could have been applied to other categories. In addition, particularly in countries without local NGO assistance, design and reporting were not captured as cash costs, but were instead part of the regular job duties of the ministry staff. Although all of the TAS measured in this survey were conducted with support from USAID and an international NGO, the costs of TAS implementation would be expected to be similar for other implementers, including MOHs alone, given that costs of international NGO technical support were not included. As national programs scale up TAS implementation, some might need to engage other local partners to aid in survey design, implementation and reporting; thus the inclusion of local NGO costs in some surveys likely reflects the reality of survey implementation and associated costs, regardless of funding source.

The lack of significant difference between costs by source of expenditure data helps support the idea that the grant mechanisms utilized in some instances by USAID are a feasible tool to provide support to national programs for survey activities. Given the due diligence necessary to prepare grant budgets, it is not surprising that no significant difference was found between grant budgets and actual expenditures. The results of this study are likely generalizable to support for TAS using other financial mechanisms which are based on detailed review of line item budgets.

The higher costs of household surveys than school-based surveys was to be expected as household surveys often take extra time to enumerate all households in a cluster and then randomly select the appropriate sample number. Furthermore, instead of one trip for a school-based survey, a household survey can require that the TAS team take two trips: one for preparations such as meeting with community leaders and identifying households and one for the survey itself. Meetings with a diverse group of community leaders also generally require more time than meetings with school leaders. The additional trip increases transportation and personnel costs, which together drive 83.5% of TAS costs.

Similarly, higher costs of surveys in Africa than Asia could be partially driven by the fact that all household surveys were in African countries. WHO guidance presumes that, in areas with low school attendance, household surveys should be conducted to ensure non-enrolled children are included in the sampling framework. Results of ongoing studies to determine whether non-enrolled children are more likely to be infected with LF than school-enrolled children will be critical to determine whether household surveys are truly necessary.

The retrospective nature of this cost study rendered some potentially useful data points impossible to gather. Expenditure reports noted total amounts spent but not quantities purchased or unit costs, which precluded an ingredients approach to costing. Furthermore, no records were available of distances travelled for each TAS. An attempt was made to collect data on person-days per survey, but the data collected on number of people involved in each survey was not available in every report, so it was excluded from the analysis. Further research, including prospective collection of person-time information, would be helpful to elucidate how survey efficiencies could be improved.

As indicated above, by 2020, WHO estimates that at least 4,671 surveys will be needed to confirm elimination of LF in endemic countries. Using the median cost of a survey (excluding RDT costs), this calculates to $44.5 million necessary to support the cost of these surveys. This projection is critical to help national programs and donors forecast support needed over the coming years to meet the global goal of elimination of LF as a public health problem and to stimulate researchers to develop greater efficiencies for the design and implementation of these surveys.
